# Supplementary material for: First Known Report of mcr-Harboring Enterobacteriaceae in the Dominican Republic
Source: Int J Environ Res Public Health. 2023 Mar 14;20(6):5123. doi: 10.3390/ijerph20065123 (PMC10049167; doi:10.3390/ijerph20065123)
Supplement: Supplementary file 1 [file ijerph-20-05123-s001.zip › ijerph-2209234-supplementary.pdf]

Table S1. Characteristics of *mcr*-positive isolates, including animal source and sample type.

Isolate ID	Animal source	Sample type	Location	Genus and species	Sequence type	*mcr* WGS Result	NCBI Short Read Archive ID
DR2-002C4	Swine	Fecal	Santo Domingo Norte	*E. coli*	1602	*mcr*-1.1	SRR17583609
DR2-003C1	Swine	Fecal	Santo Domingo Norte	*E. coli*	1602	*mcr*-1.1	SRR17583597
DR2-004A1	Swine	Fecal	Santo Domingo Norte	*E. coli*	29	*mcr*-1.1	SRR18036934
DR2-004C1	Swine	Fecal	Santo Domingo Norte	*E. coli*	1602	*mcr*-1.1	SRR17583588
DR2-004C2	Swine	Fecal	Santo Domingo Norte	*E. coli*	1602	*mcr*-1.1	SRR17583582
DR2-005C1	Swine	Fecal	Santo Domingo Norte	*E. coli*	1602	*mcr*-1.1	SRR17583576
DR2-006A1	Swine	Fecal	Santo Domingo Norte	*E. coli*	50	*mcr*-1.1	SRR17583608
DR2-006A3	Swine	Fecal	Santo Domingo Norte	*E. coli*	410	*mcr*-1.1	SRR17583591
DR2-006C1	Swine	Fecal	Santo Domingo Norte	*E. coli*	8233	*mcr*-1.1	SRR17583580
DR2-006C2	Swine	Fecal	Santo Domingo Norte	*E. coli*	8233	*mcr*-1.1	SRR17583596
DR2-006C3	Swine	Fecal	Santo Domingo Norte	*E. coli*	8233	*mcr*-1.1	SRR17583587
DR2-006C4	Swine	Fecal	Santo Domingo Norte	*E. coli*	8233	*mcr*-1.1	SRR17583581
DR2-006C5	Swine	Fecal	Santo Domingo Norte	*E. coli*	8233	*mcr*-1.1	SRR18036933
DR2-006C6	Swine	Fecal	Santo Domingo Norte	*E. coli*	8233	*mcr*-1.1	SRR17583575
DR2-007A1	Swine	Fecal	Santo Domingo Norte	*E. coli*	8233	*mcr*-1.1	SRR18036932
DR2-007C1	Swine	Fecal	Santo Domingo Norte	*E. coli*	1602	*mcr*-1.1	SRR17583574
DR2-007C2	Swine	Fecal	Santo Domingo Norte	*E. coli*	1602	*mcr*-1.1	SRR17583573
DR2-010A1	Swine	Feed	Santo Domingo Norte	*E. coli*	1771	*mcr*-1.1	SRR17583569
DR2-010A2	Swine	Feed	Santo Domingo Norte	*E. coli*	1771	*mcr*-1.1	SRR17583595
DR2-101A2	Poultry	Fecal	Moca	*E. coli*	410	*mcr*-1.1	SRR17583604
DR2-101A3	Poultry	Fecal	Moca	*E. coli*	410	*mcr*-1.1	SRR17583594
DR2-198A2	Swine	Fecal	Moca	*E. coli*	191	*mcr*-1.1	SRR17583602
DR2-272A1	Beef Cattle	Meat	Santo Domingo	*E. coli*	10	*mcr*-1.1	SRR17583601
DR2-300A1	Swine	Fecal	La Vega	*E. coli*	6778	*mcr*-1.1	SRR17583600
DR2-301C4	Swine	Fecal	La Vega	*E. coli*	48	*mcr*-1.1	SRR17583607
DR2-302C1	Swine	Fecal	La Vega	*E. coli*	48	*mcr*-1.1	SRR17583606
DR2-302C2	Swine	Fecal	La Vega	*E. coli*	48	*mcr*-1.1	SRR17583593
DR2-302C3(1)	Swine	Fecal	La Vega	*E. coli*	48	*mcr*-1.1	SRR17583586
DR2-302C3(2)	Swine	Fecal	La Vega	*E. coli*	48	*mcr*-1.1	SRR17583592
DR2-302C4	Swine	Fecal	La Vega	*E. coli*	48	*mcr*-1.1	SRR17583579
DR2-303A1	Swine	Fecal	La Vega	*E. coli*	191	*mcr*-1.1	SRR17583590
DR2-303A2	Swine	Fecal	La Vega	*E. coli*	-	*mcr*-1.1	SRR17583599
DR2-304C1	Swine	Fecal	La Vega	*E. coli*	871	*mcr*-1.1	SRR17583598
DR2-304C2	Swine	Fecal	La Vega	*E. coli*	871	*mcr*-1.1	SRR17583589
DR2-304C3	Swine	Fecal	La Vega	*E. coli*	871	*mcr*-1.1	SRR17583585
DR2-305A1	Swine	Fecal	La Vega	*E. coli*	48	*mcr*-1.1	SRR17583584
DR2-305A2	Swine	Fecal	La Vega	*E. coli*	48	*mcr*-1.1	SRR17583578
DR2-306A1	Swine	Fecal	La Vega	*E. coli*	191	*mcr*-1.1	SRR17583583
DR2-306A2	Swine	Fecal	La Vega	*E. coli*	191	*mcr*-1.1	SRR17583577
